# A Evolução do Cenário da Cardiogeriatria no Brasil: Novos Desafios para um Novo Mundo

**DOI:** 10.36660/abc.20190292

**Published:** 2020-04-06

**Authors:** Caio de Assis Moura Tavares, André Feitosa Wanderley Cavalcanti, Wilson Jacob

**Affiliations:** 1 Hospital das ClínicasFaculdade de MedicinaUniversidade de São PauloSão PauloSPBrasilUnidade de Cardiogeriatria - Instituto do Coração (InCor) - Hospital das Clínicas HCFMUSP - Faculdade de Medicina - Universidade de São Paulo, São Paulo, SP – Brasil

**Keywords:** Geriatria/tendências, Avaliação Geriátrica, Dinâmica Populacional, Cardiologia/tendências, Idoso, Assistência à Saúde, Idoso Fragilizado, Saúde para Idosos

A necessidade de nos preparamos para o atendimento da população geriátrica já é reconhecida mundialmente há duas décadas. “Nós exercemos cardiologia geriátrica?”. Assim o Dr William W. Parmley intitulou seu editorial de 1997,^1^ publicado no *Journal of the American College of Cardiology* (JACC), e considerado, por boa parte dos norte-americanos, como o ponto de partida para o desenvolvimento da Cardiogeriatria naquele continente. Apesar da existência, desde 1986, de uma sociedade (Society of Geriatric Cardiology - SGC), esta não atuava sob a tutela de nenhuma das duas grandes associações de Cardiologia americanas e carecia de maior relevância. Com o envelhecimento populacional, fenômeno mundial, o encontro do cardiologista com pacientes cada vez mais longevos havia se tornado frequente. A partir desse alerta, desenvolveram-se iniciativas para suprir a nova demanda. Um currículo online de educação continuada, elaborado entre a American College of Cardiology (ACC) e a SGC foi disponibilizado, em 2007, aos seus membros e divulgado em novo editorial, de 2008, do JACC.^2^ Em 2011, a SGC foi extinta e incorporada como Seção-Membro de Cardiologia Geriátrica da ACC.

Cabe ressaltar que, naquele período, o mundo já reconhecera a inexorabilidade do envelhecimento populacional e já percebera a importância de dotar os profissionais de uma capacitação superespecializada (a especialidade na faixa etária) e de dar a essa população cada vez mais predisposta a envelhecer, a possibilidade de ter suas demandas atendidas, conforme suas características individuais, mais do que pelas doenças que a acometiam.

No Brasil, o pioneirismo do Prof. Dr. Luís Gastão Costa Carvalho do Serro Azul, em 1982 (quatro anos antes da fundação da sociedade americana sobre o tema), iniciava a Unidade Clínica de Cardiogeriatria do Instituto do Coração (InCor), um movimento que colocou o nosso país em uma posição de vanguarda na discussão desse tema. Na década de 90, a Cardiogeriatria foi reconhecida pela Sociedade Brasileira de Cardiologia, inicialmente, como um grupo de estudos em Cardiogeriatria (GEBRAC) e, desde 2005, como um departamento (DECAGE). Em 2006 e em 2014, dois artigos reforçaram para a sociedade médica Brasileira a importância da Cardiogeriatria – o primeiro, assinado pelo Prof. Dr Maurício Wajngarten,^3^ elencou os desafios que estavam por vir e a necessidade de preparação para atendimento dessa população idosa; o segundo, assinado pelo Prof. Roberto Franken e pelo Dr. Ronaldo Fernandes Rosa,^4^ destacou o progresso pela criação do DECAGE para formação de treinamento em Cardiogeriatria, parcerias com a ACC, avanços em evidência científica gerada pelo departamento, bem como elencou habilidades essenciais para o atendimento pleno do idoso. Em conformidade com essa necessidade, a Sociedade Brasileira de Cardiologia publicou diretrizes específicas de Cardiogeriatria: a primeira em 2002;^5^a segunda, em 2010,^6^ com sua atualização em 2019,^7^ além de elencar, na I Diretriz sobre Processos e Competências para a Formação em Cardiologia no Brasil,^8^ o conteúdo necessário aos profissionais em formação para o devido atendimento de doenças cardiovasculares no paciente idoso.

## A Essência da Cardiogeriatria

Por tratar-se de advento recente e ainda não totalmente difundido, muitas vezes confunde-se a Cardiogeriatria com uma Geriatria praticada pelo cardiologista ou, simplesmente, como a Cardiologia aplicada ao paciente idoso, o que, apesar de constituir parte do âmago dessa disciplina, não representa a sua totalidade. Mais adequado seria defini-la como a integração do cuidado cardiovascular com uma abordagem adequada à idade e centrada no paciente^9^ e na sua funcionalidade – conceito já exposto previamente por nossos colegas ^3, 4^, mas que, com o passar dos anos, está tomando forma, com o uso de ferramentas específicas e pré-determinadas.

De modo objetivo, pode-se considerá-la como uma prática cardiológica, integrada aos 5 Ms da Geriatria: *medication* (foco na prescrição do absolutamente necessário, visando restringir a polifarmácia, reduzir interações e efeitos adversos, respeitando os critérios de Beers na seleção de medicações adequadas ao idoso), *mentation* (vigilância, prevenção e tratamento dos distúrbios cognitivos), *mobility *(valorização e implementação de estratégias que visem manter a funcionalidade mecânica do paciente), *multimorbidity *(abordagem do paciente não com o olhar apenas sobre o sistema cardiovascular, mas considerando-se que a ocorrência de múltiplas comorbidades é a regra, e não a exceção, nesses indivíduos) e, por último, e literalmente mais importante, *matters most* (sempre valorizar a opinião do paciente sobre os benefícios e fardos que o tratamento irá acarretar, sob a ótica da sua biografia e valores pessoais, trazendo-o para o centro das decisões). Acrescentaríamos, ainda, um sexto e último M, de multidisciplinaridade, pois o cuidado desses idosos deve estar coordenado de forma horizontal por um profissional, mas nunca isolado em sua figura, sendo importante a participação de outros especialistas e profissionais de saúde. Na Unidade de Cardiogeriatria do InCor, para todos os pacientes é realizada uma avaliação geriátrica ampla com o auxílio da ferramenta TAGA-10 (*Targeted Geriatric asssesment*) ^10, 11^

## A Evolução da Cardiogeriatria

Com o passar dos anos, os avanços médicos, científicos e das condições de vida fizeram com que esses indivíduos não somente chegassem em grande número a idades avançadas, mas também com condições físicas e expectativas para o futuro que excedem o que se observava no passado, mudando os conceitos do que seria “ficar velho”^12^. Observamos ainda que, salvo poucas exceções, as doenças cardiovasculares são doenças do envelhecimento. No século XVII, o Dr Thomas Sydenham já havia declarado que “um homem é tão velho quanto suas artérias”. De modo que o paciente típico do atendimento cardiológico é uma pessoa mais velha que carrega consigo, além de mais esperanças e anseios, também um número cada vez maior de comorbidades e deteriorações relacionadas ao envelhecimento que complicam o gerenciamento tradicional baseado em diretrizes^13^. Sabe-se, ainda, que o envelhecimento dá ao aparelho cardiovascular características diversas da juventude ^14-16^, tornando ainda mais peculiar o atendimento do idoso.

O dogma tradicional de que “depois de certa idade, o paciente é velho demais para realizar procedimentos cardíacos invasivos” não tem espaço no cenário atual. Notadamente, com o avanço tecnológico vimos juntar-se ao arsenal terapêutico em Cardiologia opções que permitiram avançar no cuidado de afecções cardiovasculares que acometem desproporcionalmente indivíduos idosos - o surgimento dos Anticoagulantes Orais Diretos, Ressincronização Cardíaca, Dispositivos de Assistência Ventricular Esquerda, TAVI e Clipes Mitrais abrem novos horizontes terapêuticos. Paradoxalmente, o risco das intervenções (clínicas ou cirúrgicas) permanece alto nos muito idosos, trazendo a avaliação geriátrica e da fragilidade como uma ferramenta útil e necessária para a decisão terapêutica, podendo inclusive identificar indivíduos que se beneficiam do procedimento e aqueles nos quais o procedimento é fútil^9, 17^.

Vivemos na era da multimorbidade: dados do Medicare mostram que, entre seus usuários, ela ocorre em 63% dos que têm entre 65 e 75 anos, progredindo com a idade, chegando a ocorrer em 83% dos usuários acima de 85 anos ^18^. Seu impacto econômico é igualmente impressionante, pois apenas 14% dos beneficiários (os que relatam 6 ou mais condições crônicas) consomem 46% do orçamento anual do programa (mais de U$ 500 bilhões) ^13^.

Os paradigmas atuais de tratamento das doenças cardiovasculares são limitados para esses pacientes. A abordagem atual para o atendimento do cardiologista é amplamente impulsionada por diretrizes de prática clínica de doença única - baseadas largamente em Ensaios Clínicos Randomizados que costumam excluir deliberadamente e sistematicamente idosos com multimorbidade: avaliam, predominantemente, desfechos duros e não consideram preservação física, cognição ou qualidade de vida associada à saúde em suas análises, que seriam muito mais relevantes na avaliação do paciente, nas últimas décadas ou anos de vida. Outra limitação para a aplicação dessas diretrizes, é que o enfoque na doença pode causar inadvertidamente efeitos prejudiciais no contexto da multimorbidade - a complexidade desse tema é tanto que, frequentemente, um tratamento acarreta o surgimento de uma nova doença ou descompensação de outra condição pré-existente ^19^.

O conceito do fenômeno das “Sliding Doors”^20^ foi proposto para descrever como, no modelo atual de atendimento, um paciente com múltiplas comorbidades tem desfechos diferentes, a depender da porta em que ele entra primeiro. Por exemplo, um paciente apresentando um câncer colorretal oculto e doença arterial coronária, **ao procurar primeiro o oncologista**, tem diagnosticada a neoplasia, realiza cirurgia/quimioterapia e durante o tratamento desenvolve insuficiência cardíaca; **ao procurar primeiro o cardiologista**, o mesmo paciente tem diagnosticada obstrução coronariana grave, é submetido à angioplastia, com uso de dupla antiagregação plaquetária e, após alguns meses, apresenta uma hemorragia digestiva significativa, sendo diagnosticado o câncer, já em fase mais avançada. Acreditamos, assim como Forman DE^19^, em um novo modelo, no qual o idoso com multimorbidades tenha seu cuidado centrado em um profissional com olhar geriátrico, que coordene de maneira horizontal os cuidados, com especialistas atuando pontualmente e sob comunicação, preferencialmente, com prontuários eletrônicos compartilhados. Em tal modelo, o paciente hipotético teria as duas doenças avaliadas e tratadas em momento oportuno.

Trata-se, pois, de um novo olhar para as enfermidades, tendo o paciente como foco principal, não apenas nos seus múltiplos componentes biológicos, mas também no seu universo biográfico, o que o transforma em único, mas nem por isso, excluído dos benefícios dos avanços tecnológicos que se mostraram eficazes em outras faixas etárias e que foram testados e comprovados também nessa fase avançada da vida.

Visto que nos foi dada a inestimável oportunidade de viver mais, seja esta também uma boa opção para viver melhor.

## Conclusão

A Cardiogeriatria é um campo em evolução, ainda em processo de formação de sua identidade e definição do treinamento fundamental e mandatório. Em apenas duas décadas, evoluímos muito: identificamos gaps no conhecimento do idoso, demos os primeiros passos em direção ao estabelecimento de um currículo em cardiogeriatria, bem como desenvolvemos ferramentas específicas para a avaliação do idoso com doença cardiovascular – passos iniciais para uma subespecilidade ainda em formação. Programas de formação clínica na área ainda são raros – ao nosso conhecimento, na América do Norte, apenas na New York University, Vanderbilt University, University of Pittsburgh - nos Estados Unidos - e McGill University - no Canadá. No Brasil, existem no InCor, Instituto Dante Pazzanese e Escola Paulista de Medicina, abertos a Cardiologistas e Geriatras. Felizmente, esse ano, temos 6 profissionais em treinamento na nossa instituição – maior número desde a abertura do programa. Que seja uma representação da evolução da cardiogeriatria e um incentivo para a longa jornada que ainda temos a frente, afinal os desafios não são poucos: i) estreitar os gaps de conhecimento para os idosos; ii) ampliar a participação dos idosos incluídos nos estudos clínicos; iii) avaliar desfechos que sejam relevantes para o paciente – cognição e qualidade de vida; iv) ampliar a capacidade de formar profissionais com treinamento específico em cardiogeriatria.
