# Supplementary material for: A Evolução do Cenário da Cardiogeriatria no Brasil: Novos Desafios para um Novo Mundo
Source: Arq Bras Cardiol. 2020 Apr 6;114(3):571–3. [Article in Portuguese] doi: 10.36660/abc.20190292 (PMC7792715; doi:10.36660/abc.20190292)
Supplement: Translation

# Translation: The Evolving Landscape of the Geriatric Cardiology Field in Brazil: New Challenges for a New World

**Authors:** Caio de Assis Moura Tavares, André Feitosa Wanderley Cavalcanti, Wilson Jacob

**Affiliations:** 1 BrazilUnidade de Cardiogeriatria - Instituto do Coração (InCor) - Hospital das Clínicas HCFMUSP - Faculdade de Medicina - Universidade de São Paulo, São Paulo, SP – Brazil

**Keywords:** Geriatrics/trends, Geriatric Assessment, Population Dynamics, Cardiology/trends, Aged, Delivery of Health Care, Frail Elderly, Health Services for the Aged

“Do we practice Geriatric Cardiology?”. It was with this editorial, published in the *Journal of the American College of Cardiology* (JACC),^1^ in 1997, that Dr. William W. Parmley highlighted the need to develop specialized care for the geriatric population worldwide. Two decades later, it is considered by some as the starting point for the growth of Geriatric Cardiology in America. Even though the Society of Geriatric Cardiology was founded in 1986, it was not bonded to either the American College of Cardiology or the American Heart Association, and it lacked major impact in the field. With the global phenomenon of aging, cardiologists were involved in the care of elderly patients more frequently, thus initiatives were developed to fulfill their needs. For instance, in 2007, an online curriculum of continuing medical education (CME) was developed by the ACC/SGC and provided for its members, as well as published in a new editorial in the JACC.^2^ In 2011, the SGC was extinguished and added to the Geriatric Cardiology chapter of the ACC.

By that time, the world had already recognized the inevitability of population aging and had already realized the importance of both giving professionals a super specialized competence (specialization in this age group) and of providing the potentially aging population with the possibility of having their demands answered by taking into account their individual characteristics, rather than the conditions they suffered from.

In Brazil, under Prof. Dr. Luís Gastão Costa Carvalho do Serro Azul’s pioneering leadership, the Heart Institute (InCor) Cardio Geriatric Clinical Unit was founded in 1982 (4 years before the foundation of the American Society of Geriatric Cardiology), a movement that placed our country at the forefront of this issue. In the 90s, Geriatric Cardiology was recognized by the Brazilian Cardiology Society, initially as a study group in Cardiogeriatrics (GEBRAC) and, since 2005, as a department (DECAGE). In 2006 and 2014, two articles were published which reinforced the importance of Geriatric Cardiology among the Brazilian medical society. The first article, by Prof Dr. Maurício Wajngarten,^3^ listed the challenges ahead and the need for preparing for the elderly population care. The second article, by Prof Roberto Franken and Dr. Ronaldo Fernandes Rosa,^4^ highlighted the contribution of the DECAGE for the education and training in Geriatric Cardiology, by means of a partnership with the ACC, as well as the advances in scientific evidence generated by the department. In addition, it listed essential abilities for full care of the elderly. Due to those needs, the Brazilian Cardiology Society published specific Geriatric Cardiology guidelines: the first, in 2002,^5^ and the second, in 2010,^6^ which was updated in 2019.^7^ The necessary contents of training professionals for due care of cardiovascular disease in elderly patients were also listed in the 1st Guidelines on Processes and Competences for Cardiology Training in Brazil.^8^

## The essence of geriatric cardiology

Because it is a recent and not widespread specialty, Geriatric Cardiology is often confused with Geriatrics that is practiced by a cardiologist or simply with Cardiology that is applied to elderly patients. Although this is part of the discipline’s core, it does not represent the whole picture. It would be more adequate to define it as integrated and age-adequate cardiovascular care, centered on the patients^9^ and their functionalities – a concept previously presented by our colleagues^3,4^- that has been evolving and taking shape, as a result of the use of specific and predetermined tools.

Objectively, one could consider it as cardiology practice integrated with the Geriatric’s 5 Ms: medication (focusing on prescribing the absolutely necessary, targeting at reducing polypharmacy, minimizing interactions and adverse reactions; following Beers criteria to select appropriate medication for the elderly), mentation (vigilance, prevention and treatment of cognitive disturbances), mobility (valuing and implementing strategies that seek to maintain the patient’s mechanical functionality), multimorbidity (approaching the patient not only by looking up the cardiovascular system, but also by considering the occurrence of multiple comorbidities to be the rule, not the exception, in these individuals), and last, but literally not least is matters most (always consider the patient’s opinion regarding the benefits and burdens of the treatment, taking into account the biography and personal values, bringing the patient into the center of decision making). We would also add a sixth and last M, multidisciplinary, for the care of the elderly, remembering it must be coordinated in a horizontal fashion by a professional, but never concentrated in only one person, giving due importance to the participation of other specialists and healthcare professionals. At our institute, in the Geriatric Cardiology Unit, we perform in all patients a Comprehensive Geriatric Assessment with the aid of the 10-TaGA (10-Minute Targeted Geriatric Assessment) tool.^10,11^

## Trends in geriatric cardiology

Over the years, medical, scientific and life conditions advances have caused individuals to reach advanced ages in large numbers, in physical conditions and future expectations that exceed what was once observed, changing concepts of what “getting old” means.^12^ We observe that, save for a few exceptions, cardiovascular diseases are aging-related diseases. In the 17th century, Dr Thomas Sydenham had already declared that “a man is as old as his arteries”. Therefore, the typical cardiology patient is an older person who has, beyond more expectations and aspirations, also an increasingly number of comorbidities and age-related deteriorations that complicate traditional guideline directed management.^13^ It is also known that aging provides the cardiovascular system with characteristics diverse from those of young people,^14-16^which makes the care of the elderly even more unique.

The traditional dogma that “after a certain age, the patient is too old for being submitted to invasive cardiac procedures” has no longer room in the current scenario. Notably, with the technological progress, we have seen the joining of the cardiologist’s therapeutic arsenal options that have allowed for the care of cardiovascular affections that disproportionally affect the elderly. The forthcoming of Direct Oral Anticoagulants, Cardiac Resynchronization Therapy, Left Ventricular Assist Devices, TAVR and Mitral Clips open up new therapeutic horizons. Paradoxically, interventional risks (clinical or surgical) remain high in the very elderly, making geriatric and frailty evaluation useful and necessary tools for therapeutic decision making, including distinguishing those individuals who might benefit from a certain procedure from those who will not.^9,17^

We live in a multimorbidity age: Medicare data shows that, among its users, it occurs in 63% of those between 65 and 75 years old, progressing with age until it occurs in 83% of users over 85 years of age.^18^ Its economic impact is equally impressive, for only 14% of beneficiaries (those who report 6 or more chronic conditions) consume 46% of the programs annual budget (over $500 billion).^13^

Current treatment paradigms for treating cardiovascular disease are limited for elderly patients. Usual approach for cardiological care is widely driven by clinical practice single disease guidelines – largely based on Randomized Clinical Trials that often deliberately and systematically exclude elderly patients with multimorbidity; they evaluate predominantly hard endpoints and do not consider physical preservation, cognition or life quality associated with health in their analysis, which would be much more relevant for evaluating the patients in their last decades or years of life. Another limitation for applying those guidelines is that focus on disease may inadvertently cause harmful effects in the multimorbidity context – this issue is extremely complex, since a treatment often entails the emergence of a new disease or decompensation of another preexisting condition.^19^

The Sliding Doors^20^ phenomenon was proposed to describe how, in the current model of care, patients with multiple comorbidities may have different outcomes, depending on the door through which they go first. For example, a patient with an occult colorectal cancer and coronary artery disease, by going first to an Oncologist, is diagnosed with neoplasia, goes under surgery/chemotherapy and during treatment develops heart failure; by going first to a Cardiologist, the same patient has a severe coronary obstruction diagnosed, has an angioplasty made, uses dual antiplatelet therapy and, after a few months, presents significant gastrointestinal bleeding, and is diagnosed with cancer at a more advanced stage. We believe, as Forman DE,^19^ in a new model: in which multimorbidity elderly patients care is centered on one professional with a geriatric point of view, who coordinates the care in a horizontal fashion, with specialists acting punctually and under communication, preferably with shared electronic medical records. In such model, our hypothetical patient would have had both diseases evaluated and treated in an opportune moment.

It is, indeed, a new look on illness, with the patients as the primary focus, not only with their multiple biological components, but also within their biography, which makes each of them unique, but not really excluded from the benefits of technological advances that have proven effective for other age groups and were also tested and proven in this advanced life stage.

Since we were given the invaluable opportunity of living longer, may it also be an option for a better life.

## Conclusion

Geriatric Cardiology is an evolving field, still in the process of forming its identity and defining which training is mandatory and fundamental. In a couple of decades only, we have evolved a lot. Gaps within the knowledge of the elderly were identified, we took the first steps to establish a Geriatric Cardiology curriculum and develop specific tools for evaluating the eldery with cardiovascular diseases – initial steps for a subspecialty that is still in the making. Formal clinical training is still rare – to our knowledge, in North America, it is only offered in New York University, Vanderbilt University, University of Pittsburgh – in the United States – and McGill University – in Canada. In Brazil, we have fellowships at the InCor (Heart Institute), Instituto Dante Pazzanese and Escola Paulista de Medicina, open to Cardiologists and Geriatricians. Gladly, this year we have 6 professionals under training in our institution – the greatest number since the program was opened. We expect that this represents the evolution of Geriatric Cardiology and an incentive on the long journey we have ahead. After all, the challenges are not few: i) narrowing the gaps on the knowledge of the elderly; ii) increase the participation of the elderly included in clinical trials; iii) evaluate endpoints that are relevant for our patients – cognition and quality of life; iv) increase the ability to form professionals with specific training in Geriatric Cardiology.
